# Development of an Instrumented Glove for Palmar Pressure Assessment in Kayakers

**DOI:** 10.3390/s26123966

**Published:** 2026-06-22

**Authors:** Corentin Depontailler, Gurvan Jodin, Corentin Porcon, Clémence Alglave, Antoine Marin, Florence Razan

**Affiliations:** 1 Department of Mechatronics, École Normale Supérieure de Rennes, 35170 Bruz, France; gurvan.jodin@ens-rennes.fr (G.J.); florence.razan@ens-rennes.fr (F.R.); 2 Université de Rennes, ENS Rennes, CNRS, IETR—UMR 6164, 35170 Bruz, France; 3 Movement, Sports and Health (M2S) Laboratory, EA 7470, Université Rennes 2, ENS Rennes, 35170 Bruz, France

**Keywords:** flexible sensors, low energy, smart garment, wearable sensors, hand pressure, palm mapping, kayak paddle, sport monitoring

## Abstract

Understanding hand–paddle interaction is essential for optimizing performance and preventing injury in kayaking, yet coaches still lack objective, practical tools. We present a soft, instrumented glove that measures and dynamically maps palmar pressure throughout the stroke cycle. A matrix of piezoresistive sensors is integrated into the glove and connected to dedicated electronics housed in a waterproof enclosure. A viscoelastic model converts sensor resistance into forces, enabling time-resolved 3D mapping of contact mechanics. Data are transmitted via Bluetooth Low Energy (BLE). Experimental validation on a kayak ergometer across multiple cadences demonstrated accurate measurements (per-sensor root mean square error (RMSE) of ±2 N), clear delineation of pull and push phases, evolving pressure distribution over the motion, and a peak total right-hand force of 186 N at high cadence. Beyond feasibility, these results position the glove as a practical training aid: it supports athlete-specific load monitoring and the early detection of potentially problematic movement patterns.

## 1. Introduction

### 1.1. General Context

Measuring hand grip forces is a central issue in the study of hand–object interactions, particularly in sport and health. In public health, grip strength is commonly assessed as the maximal isometric force exerted when squeezing a dynamometer. Lower grip strength has been associated with increased risks of all-cause and cardiovascular mortality [1], while the screening of sarcopenia and frailty remains highly dependent on the thresholds used to define low grip strength [2]. International reference standards have further highlighted the importance of harmonized measurement protocols to facilitate comparisons across populations and contexts [3]. In addition, absolute grip strength appears to outperform several normalized indices for mortality prediction [4]. Despite their clinical relevance, these approaches rely on standardized maximal-grip measurements and provide only limited information about real-world palmar grasp, palm–finger coordination, grip technique, or the evolution of force during movement.

In sport, grip strength is both a capacity to be preserved and an indicator of fatigue. For example, warm-up strategies can influence sport-specific performance while affecting grip strength differently in young judoka [5]. In climbing, elite athletes exhibit superior intermittent endurance of the finger flexors and faster muscle reoxygenation during repeated efforts, emphasizing the importance of the forearm musculature in sustaining prolonged holds [6]. However, these studies remain largely based on standardized gripping tasks or specific finger postures and therefore only partially reflect the complexity of real hand–object interactions, where palm–finger integration, object geometry, friction, and force distribution play a central role.

### 1.2. Sensor Technologies

Palmar pressure can be measured using several sensing technologies. Piezoresistive approaches based on low-cost materials such as Velostat^®^ have been integrated into tactile gloves for pressure sensing and finger motion tracking [7,8,9,10]. These systems provide detailed information on hand–object interactions but often require complex wiring architectures and generally rely on local pressure measurements rather than direct estimation of global grip force. More advanced piezoresistive sensors capable of measuring both normal and shear forces have also been proposed [11], although their integration into wearable systems remains limited. Force-sensing resistors (FSRs) are widely used because of their ease of integration and lightweight instrumentation [12,13,14], but they typically exhibit nonlinear responses and mainly measure normal loads. To improve robustness, some studies combine FSR with electromyography (EMG) and kinematic measurements [15], resulting in more complex acquisition systems. Alternative technologies include capacitive sensors, which can measure normal and shear stresses but generally require more complex electronics [16]; piezoelectric sensors based on polyvinylidene fluoride (PVDF), which are effective for vibration and slip detection but less suitable for sustained pressure measurements [17]; and optical fiber systems used primarily for finger kinematics assessment [18], whose deployment is limited by cost and fragility. Finally, smart textiles have attracted increasing interest because their flexibility and ease of integration into garments enable continuous physiological monitoring [19].

### 1.3. Sport Context: The Case of Kayaking

Dynamic settings such as kayaking raise three specific challenges: a cylindrical contact surface, a highly dynamic movement context, and a wet environment. These constraints make direct measurement of hand forces difficult, especially in real on-water conditions.

Piezoresistive technology is the predominant approach in this area. The earliest attempts to instrument kayaking movements relied on strain gauges mounted on key elements of the boat or simulator [20,21]. In Sturm et al. [22], a wireless system was proposed to capture forces on both the paddle and the footrest. Data were transmitted via Bluetooth, and ergometer tests revealed a coherent force profile between the upper and lower limbs. While discreet and portable, the system is essentially confined to laboratory use and does not measure pressures directly at the hands. The same limitation is observed in similar studies [23,24].

Subsequently, Bonito et al. [25] analyzed the forces generated by elite kayakers at the paddle and footrests using strain gauges. Their study highlights a correlation between force profiles and dynamic performance on an ergometer. They clearly show that forces applied to the footrests reach values roughly two to three times higher than those measured at the paddle, with peaks exceeding 700 N versus an average of about 240 N at the hand. However, pressure exerted specifically by the fingers or the palm is not captured, limiting fine-grained analysis of grasp. In addition, the system remains relatively cumbersome due to the conditioning electronics required.

A more advanced approach was proposed by Nates [26], who designed a comprehensive three-dimensional system measuring mechanical loads along the longitudinal, transverse, and vertical axes on the paddle while also integrating measurements at the seat and foot supports. The force sensors are based on calibrated strain gauges to detect asymmetric contributions from the hands. In parallel, an optoelectronic motion-capture system with 101 markers placed on the athlete and the ergometer was used to reconstruct gesture kinematics in detail. Although effective and validated in real conditions, the setup is wired and bulky and requires rigorous calibration, which limits autonomous use by coaches and athletes.

Commercial systems dedicated to kayaking primarily focus on measuring deformation of the paddle shaft, providing information on cadence, orientation during movement, and shaft flexion [27]. However, none of these systems explicitly accounts for palmar pressure. Instrumented glove systems available on the market are listed in Table 1.

None was designed specifically for kayaking or for fine, localized measurement of hand pressure in a dynamic, aquatic environment. The major limitations in water-sport contexts are system waterproofing [28,29,30,31,33] and insufficient battery life to cover full training sessions [34]. Some gloves are too thick or bulky, reducing freedom of movement. Most systems target rehabilitation [18,35,36,37], robotics [13,38], or ergonomic research [34,39,40], and they do not meet the economic and functional constraints of outdoor athletic use. Wrist and forearm-worn devices provide promising approaches for estimating physical effort from proximal measurements, for example, using force myography or EMG combined with inertial sensing. However, these approaches provide indirect estimates of interaction forces and do not measure contact pressure at the palm object interface or its spacetime distribution, which requires pressure-sensing instrumentation located on the hand, such as on the palm or fingertips [41,42].

Our previous study [27] mainly focused on the characterization of flexible Velostat-based piezoresistive sensors with a regular matrix of identical sensors, applied to a non-planar surface using a symmetric sensor arrangement that did not allow the estimation of global palmar pressure. In contrast, the present work investigates the integration of these sensors into a functional textile support while exploring different sensor shapes and sizes for comprehensive palmar pressure analysis during gripping tasks. This study demonstrates the feasibility of an instrumented textile glove embedding sewn flexible piezoresistive sensors to measure palmar pressure distribution under dynamic conditions. The proposed device is specifically applied to the context of kayaking, where performance strongly depends on biomechanical and physiological parameters related to the hand–paddle interaction. Beyond kayaking, this approach also opens perspectives for other sports applications as well as medical applications, particularly for rehabilitation monitoring following injury or surgery.

## 2. Materials and Methods

### 2.1. System Description

**Figure 1:**
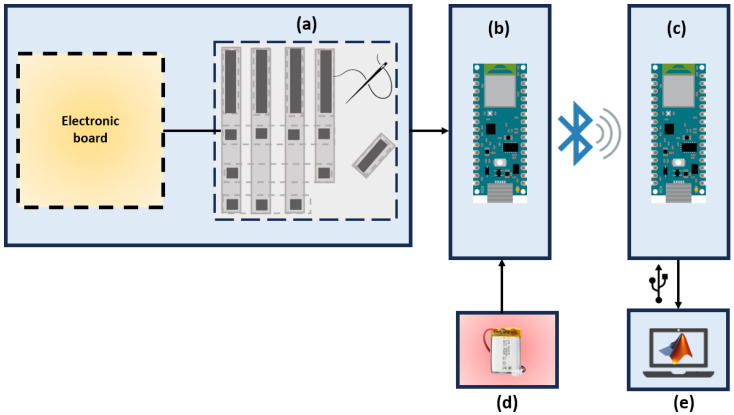
System illustration: (**a**) textile glove with sensor matrix; (**b**) peripheral device; (**c**) central unit; (**d**) battery; (**e**) processing on PC.

The developed system is an instrumented glove incorporating flexible piezoresistive sensors based on a carbon-loaded polyethylene composite: Velostat^®^ (3M Electronics Division, Saint Paul, MI, USA) [43]. Sensor outputs are acquired by an Arduino Nano 33 BLE microcontroller (Arduino, Ivrea, Italy), referred to as the “peripheral”, which reads resistance variations at each node of the sensor matrix described in Section 2.2. The peripheral is powered by a 3.7 V, 400 mAh LiPo battery (Model 502535, EAST (Shenzhen) Technology Co., Ltd., Shenzhen, China). This development board was selected to meet the system’s energy constraints and to ensure sufficient autonomy over full training sessions through the use of the BLE protocol. The peripheral transmits data wirelessly to a second Arduino Nano operating as a BLE “central,” which is connected to the PC. Data processing is then performed using MATLAB R2025a (MathWorks, Natick, MA, USA). An overview of the system is shown in Figure 1.

### 2.2. Choice of the Number of Pressure Points to Measure

To determine how many sensors to place on the palmar side, it is essential to understand the hand’s architecture and the muscular loads engaged when gripping a cylinder.

Intrinsic muscles provide fine modulation of pressures, stabilize the thumb, and supply lateral support, thereby increasing the contact area. Their action enables the redistribution of the forces generated by the extrinsic muscles. Consequently, for meaningful data acquisition, sensors should be placed primarily on the fingers, the thenar and hypothenar eminences, and along the lateral contact zones of the hand, where synergies between extrinsic and intrinsic muscles are expressed during gripping [44].

The study by [45] showed that during a power grip, the principal contact regions are located at the distal phalanges, the palm near the radius, and the thenar and hypothenar eminences, with a large contact area that depends on hand size and handle diameter. These zones, therefore, concentrate the main pressures during grasping.

As reported in [46], gripping a cylinder engages the entire palm and fingers, unlike a finer pinch grip. It also describes that pressures are localized on the palmar surface of the hand and that load distribution is influenced by soft tissues, such as digital pads and palmar pulp, which deform under pressure.

**Figure 2:**
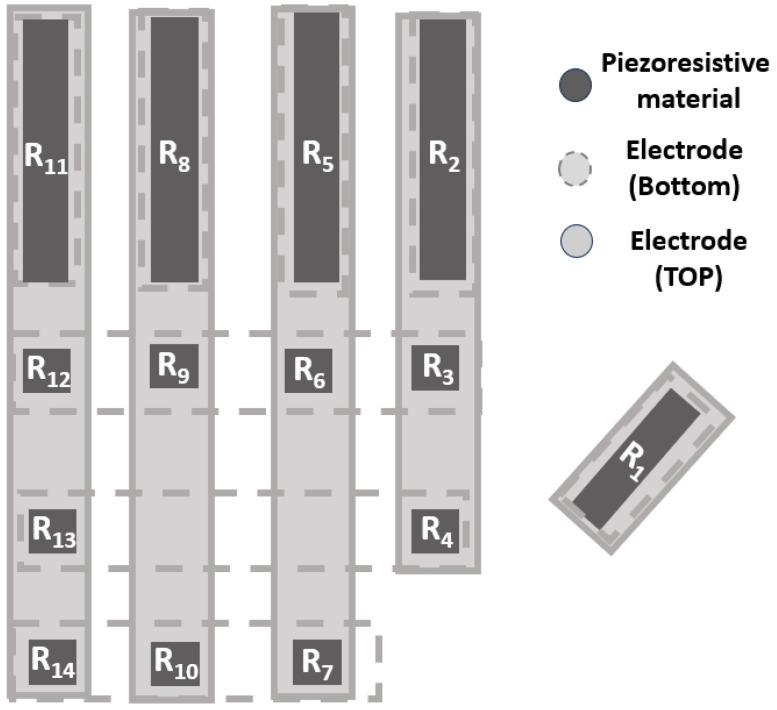
Sensor matrix selected for the right hand: Palmar view.

Accordingly, the sensor matrix comprises 14 measurement points strategically distributed over the palmar surface of the hand. Each point is associated with a variable resistor R_i_ with i ∈ [1, 14]. Each finger is equipped with a 7 cm^2^ sensor to capture the overall pressure exerted by all phalanges. In addition, nine 1 cm^2^ sensors are positioned on the palm: two on the thenar eminence, two on the hypothenar eminence, one on each of the four metacarpal heads, and one at the carpal tunnel. This arrangement is designed to capture both the primary support zones and the stabilization points. The diagram in Figure 2 illustrates this configuration.

### 2.3. Glove Fabrication

**Figure 3:**
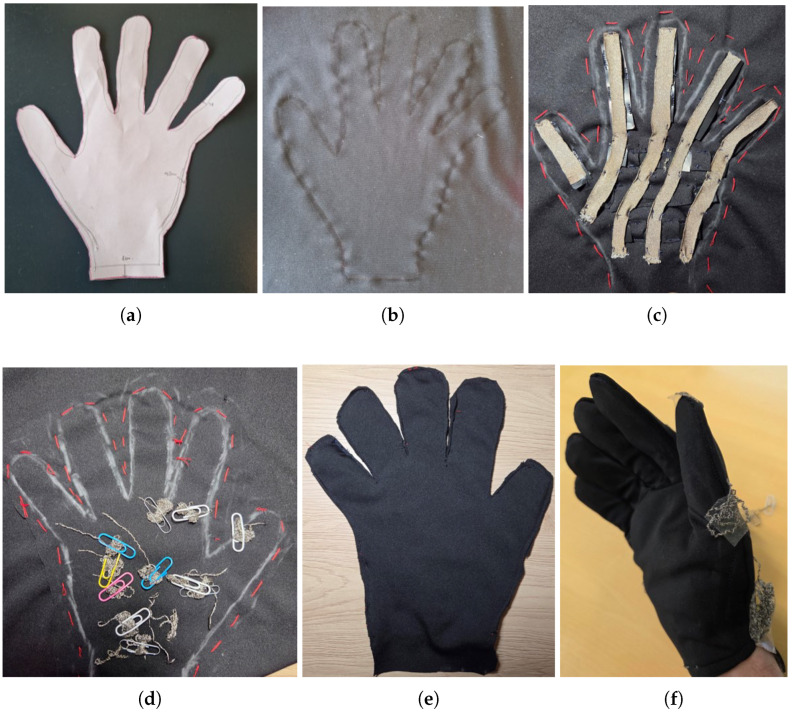
Glove manufacturing steps: (**a**) right-hand pattern; (**b**) tracing on fabric; (**c**) piezoresistive sensor matrix (palmar view); (**d**) dorsal view of the glove; (**e**) cutting out the glove; (**f**) view of the glove being worn.

The first step is to create the hand pattern (Figure 3a). Once the pattern outline is transferred onto the fabric using a fabric marker (Figure 3b), the lower-layer electrodes and the piezoresistive material are stitched in a straight stitch with polyester thread. The goal is to avoid damaging the material so as to preserve its properties. Next, the upper-layer electrode strips are positioned (Figure 3c). The conductive leads can then be routed to the opposite side of the glove (Figure 3d). The conductor is a multi-strand, silver nano-plated thread designed to integrate electronic components into textiles [47]. The glove is then closed (Figure 3e) with a lining to prevent any contact between the skin and the electrodes and finally turned right side out (Figure 3f) so that the leads exit on the outside.

**Figure 4:**
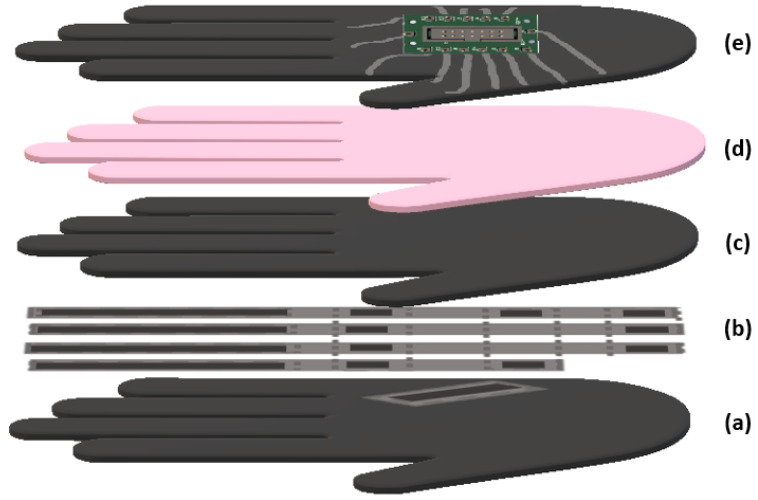
Cross-section view of the glove: (**a**) palmar view; (**b**) sensor matrix; (**c**) fabric lining; (**d**) hand; (**e**) dorsal view (wires and PCB).

A cross-sectional view of the glove is provided in Figure 4. It should be noted that this glove prototype was manufactured using the anthropometric measurements of a single reference user due to participant availability at the time of fabrication tests. As a result, the current device is size-specific. It can be worn by other individuals provided their hand dimensions fall within the same size range.

An electronics board with an insulation displacement connector (IDC) is sewn onto the dorsal side of the glove (Figure 4e) to collect data from each node of the matrix. This connector is then linked to the enclosure described in Section 2.5.

### 2.4. Sensor Characterization

This section presents the different characterization procedures implemented to evaluate the sensor performance. A static characterization was first performed directly on the sensor matrix prior to glove closure. This was followed by a dynamic characterization with the glove worn, allowing the assessment of the sensor behavior under realistic operating conditions. Since the contact between the skin and the sensor induces a decrease in resistance, a new baseline resistance was established when the glove was worn and measured before conducting the dynamic tests.

#### 2.4.1. Static Characterization

**Figure 5:**
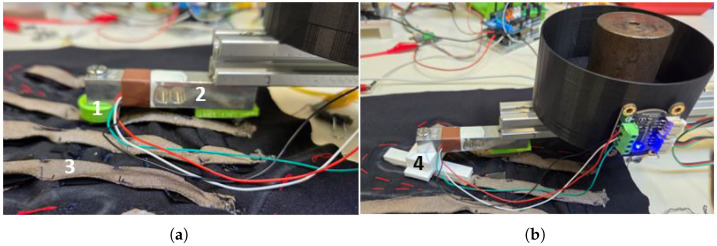
Static sensor characterization: (**a**) 1 cm^2^ sensor; 1. PLA support 1 cm^2^; 2. strain gauge; 3. piezoresistive sensor matrix; (**b**) 7 cm^2^ sensor; 4. PLA support 7 cm^2^.

The sensor matrix was characterized under static conditions using a test bench to observe the sensor’s reproducible behavior under load and to verify repeatability [27]. The experimental protocol consisted of applying a mass to the sensor for 50 s, corresponding to the material’s stabilization time. The mass was then removed, and the sensor was left at rest for 1 min before placing a new mass. This procedure was repeated five times on each sensor to ensure measurement reproducibility. Note that the sensors differ in size and therefore must be characterized independently. Specifically, the finger sensors have an area of 7 cm^2^ (Figure 5b), while the palm sensors are 1 cm^2^ (Figure 5a), which requires two separate 3D-printed polylactic acid (PLA) fixtures to distribute load uniformly over each sensor. This process was carried out for all nodes of the sensor matrix.

In addition, the sensitivity and hysteresis of the sensors were investigated. Owing to the nonlinear resistance–pressure relationship, the sensitivity was evaluated separately over two pressure intervals: 200 to 500 kPa and 550 to 1100 kPa. For each interval, the sensitivity was calculated according to Equation (1).

(1) $$S_{i,j}=\frac{G_{j}-G_{i}}{P_{j}-P_{i}}$$

where $G_{i}$ and $G_{j}$ are the conductance values measured at pressures $P_{i}$ and $P_{j}$, respectively. The sensitivities corresponding to the lower and higher pressure intervals are denoted as $S_{low}$ and $S_{high}$, respectively.

Hysteresis was quantified by comparing the loading and unloading curves obtained during the calibration procedure and was calculated using Equation (2).

(2) $$\mathrm{Hysteresis}\left(\%\right)=\left|\frac{R_{\mathrm{increasing}}-R_{\mathrm{decreasing}}}{R_{\max}-R_{\min}}\right|\times 100$$

where $R_{\mathrm{increasing}}$ and $R_{\mathrm{decreasing}}$ denote the resistance values measured during the loading and unloading processes, respectively, at the same applied force, while $R_{\max}$ and $R_{\min}$ represent the maximum and minimum resistance values recorded over the entire cycle. After completion of the loading phase, the unloading phase was performed by sequentially removing the applied masses while maintaining a stabilization period of 50 s between successive load levels. The resulting loading and unloading responses were subsequently used for hysteresis evaluation.

#### 2.4.2. Dynamic Characterization

**Figure 6:**
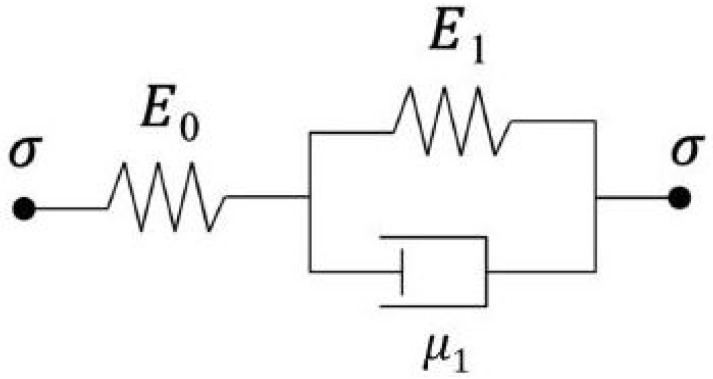
Viscoelastic model of the sensor. σ is the mechanical stress, $E_{0}$ and $E_{1}$ are the Young’s moduli of the elastic springs, and $\mu_{1}$ is the viscosity coefficient of the dashpot [27].

Dynamic characterization was performed to estimate the force applied to the sensor matrix from the resistance variations measured at each sensing node. This approach relies on the inverse viscoelastic model proposed by Laaraibi et al. [27], based on a standard linear solid representation. As shown in Figure 6, the model consists of an elastic spring of Young’s modulus $E_{0}$ connected in series with a Kelvin–Voigt element, composed of a spring of Young’s modulus $E_{1}$ in parallel with a dashpot of viscosity coefficient $\mu_{1}$.

In this model, the mechanical stress σ corresponds to the force applied to the sensor normalized by the active sensing area according to Equation (3).

(3) $$\sigma =\frac{F}{S_{\mathrm{sensor}}}$$

where *F* is the applied force in newtons and $S_{\mathrm{sensor}}$ is the active surface area of the considered sensor in square meters.

A nonlinear constitutive relationship links the sensor resistance to the strain of the viscoelastic model according to Equation (4).

(4) $$R=R_{0}\left(1-\varepsilon \right)e^{-\theta \varepsilon }$$

where *R* is the sensor resistance, $R_{0}$ is the initial resistance, ε is the strain, and θ is a relaxation parameter governing the nonlinear resistance–strain relationship. The strain was then used within the viscoelastic model to estimate the corresponding stress and applied force.

The model parameters were identified by minimizing the error between the estimated force and the reference force measured by the test bench. The optimized parameters are the Young’s moduli $E_{0}$ and $E_{1}$ and the viscosity coefficient $\mu_{1}$. In the inverse formulation, the mechanical properties are expressed as load-dependent quantities. The corresponding coefficients ($E_{0a}$, $E_{0b}$, $E_{1a}$, $E_{1b}$, $\mu_{1a}$ and $\mu_{1b}$) were identified experimentally for each sensor geometry. The model parameters can be expressed according to Equations (5)–(7).

(5) $$E_{0}=E_{0a}\;\cdot \;F+E_{0b}$$

(6) $$E_{1}=E_{1a}\;\cdot \;F+E_{1b}$$

(7) $$\mu_{1}=\mu_{1a}\;\cdot \;F+\mu_{1b}$$

The complete formulation of the viscoelastic model and its parameter identification procedure have been detailed in a previous study [48] and are therefore only briefly summarized here.

Since contact between the skin and the sensor modifies the initial electrical state by decreasing the resistance, a new baseline resistance $R_{0}$ was first measured after the glove was donned. This value was then used as the reference state for all dynamic measurements.

Dynamic loading tests were performed by applying the test bench directly to the gloved hand. Each trial provided a dataset composed of resistance, force, and time measurements. These data were used to locally optimize the model parameters at each sensing node. Once identified, the calibrated model was used to estimate the applied force point by point across the sensor matrix.

#### 2.4.3. Durability and Recovery Test Protocol

To assess the long-term stability and durability of the sensor node, an additional cyclic loading experiment was conducted on sensing point R7. The objective was to evaluate whether repeated mechanical loading induces permanent changes in the electrical response of the sensor and to determine its ability to recover after a resting period. A constant load of 294.3 kPa was selected for this experiment, as it is representative of the force range encountered in the intended kayaking application and corresponds to the operating conditions investigated throughout this study.

The experiment consisted of successive loading and unloading phases followed by a recovery period. During the entire test, the sensor resistance was continuously recorded.

The test sequence was defined as follows:

1. Repeat the complete sequence three times:
  (a) Apply 1000 loading cycles:
    - Apply the 294.3 kPa load and maintain it for 5 s.
    - Release the load and maintain the unloaded state for 5 s.
  (b) Remove the load completely and allow the sensor to recover for 2 h.

The loading and unloading durations were selected to reproduce repeated mechanical solicitations while enabling the evaluation of cumulative effects over a large number of cycles. The 2-h recovery period was introduced to investigate the reversibility of the sensor response and to identify possible permanent drift mechanisms.

Throughout the experiment, the electrical resistance of the sensing element was monitored continuously, allowing both the short-term response during cyclic loading and the long-term evolution of the baseline resistance to be analyzed. The results of this experiment are presented in Section 3.2.

### 2.5. Experiment on an Ergometer

An embedded system was designed, comprising electronic boards that interface the sensor matrix with the conditioning electronics housed in a PLA enclosure. The conditioning electronics are enclosed in an IP65-rated waterproof housing, allowing the system to be worn around the forearm while ensuring protection of the internal electronics and full freedom of movement for the user. Although the glove itself is not waterproof at this stage of development, the current design is suitable for use on an ergometer and other dry-environment testing conditions.

**Figure 7:**
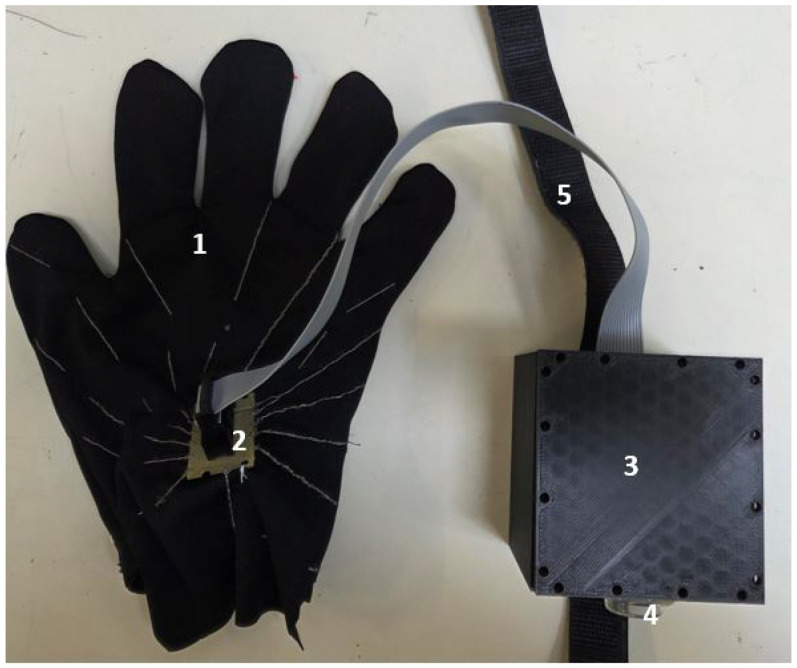
Photo of the measurement system: 1. glove; 2. electronic board; 3. enclosure; 4. switch; 5. strap.

Figure 7 shows the instrumented glove and its enclosure.

**Figure 8:**
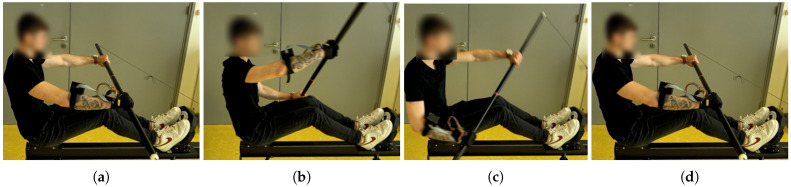
Phases of the paddling cycle reproduced during the experiment: (**a**) start of the cycle; (**b**) pushing phase; (**c**) pulling phase; (**d**) end of the cycle.

The protocol is based on a movement sequence reproducing a complete paddling cycle [49]. It begins with a 10-s static catch position, followed by 20 s at a moderate cadence of 35 strokes/min, then 20 s at a higher cadence of 48 strokes/min. Figure 8 illustrates the main hand phases observed during the cycle for the instrumented hand: the initial catch position (Figure 8a), the push phase (Figure 8b), the pull phase (Figure 8c), and the return to the initial position (Figure 8d). The initial catch position corresponds to a steady gripping condition in which the athlete is already holding the paddle. This state serves as a reference baseline for the subsequent analysis of pressure redistribution during the paddling cycle. The pressure measured in this configuration is considered an offset associated with the static grip force required to hold the paddle and is subtracted from subsequent measurements. Consequently, the reported pressure variations represent only the additional pressure exerted on the paddle throughout the paddling motion. The tests were conducted on a single user. Experimental constraints, as well as the glove’s specific measurements, did not allow tests to be carried out on other subjects. However, the aim of this study is to provide a proof of concept for the prototype presented here.

## 3. Results

This section presents the main results of the static and dynamic characterization of the piezoresistive sensors. It also details the palmar pressure mapping at each node of the matrix.

### 3.1. Static Characterization

**Figure 9:**
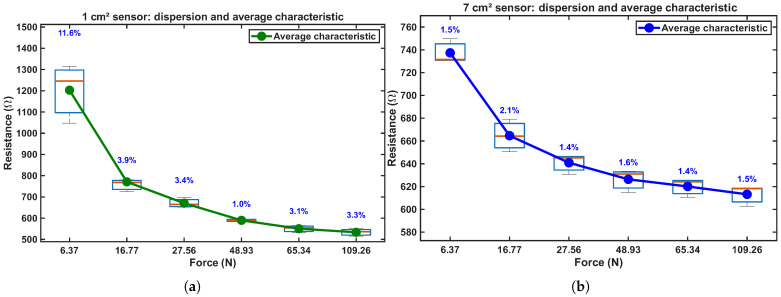
Graphs of the characteristics obtained for the two sensor sizes: (**a**) 1 cm^2^ sensor; (**b**) 7 cm^2^ sensor. Blue percentages indicate the dispersion between repeated measurements at each force level. Boxplots show the median (central line), interquartile range (box), and data spread (whiskers).

Figure 9a,b show the mean resistance as a function of the applied load for a 1 cm^2^ sensor (mounted on the palm) and a 7 cm^2^ sensor (mounted on the fingers), together with the associated dispersion across five trials conducted in accordance with the protocol described in Section 2.4. The sensitivity and hysteresis values obtained from the static characterization are summarized in Table 2.

### 3.2. Dynamic Characterization

Table 3 summarizes the coefficients identified for the two sensor geometries. These coefficients define the load-dependent evolution of the mechanical properties used by the viscoelastic model.

**Figure 10:**
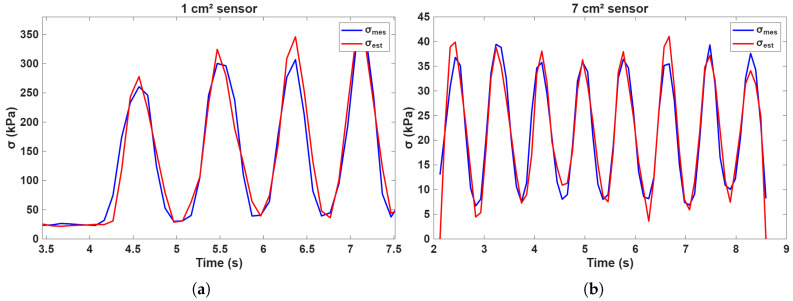
Results of the model on the optimized-parameter dataset. Comparison between the measurement ($\sigma_{\mathrm{mes}}$) and the model estimate ($\sigma_{\mathrm{est}}$): (**a**) 1 cm^2^ sensor; (**b**) 7 cm^2^ sensor.

Figure 10a,b show the measured and estimated mechanical stress as a function of time for the two sensor sections embedded in the glove. $\sigma_{\mathrm{mes}}$ corresponds to the reference force measured by the commercial force sensor visible in Figure 5 during dynamical applied force. $\sigma_{\mathrm{est}}$ is the estimated force from the viscoelastic model applied to the piezoresistive resistance measures.

The accuracy of both models was evaluated using several indicators: the root mean square error (RMSE) in Equation (8), the coefficient of determination $R^{2}$ in Equation (9), and the mean relative error over the entire signal in Equation (10). These metrics quantify how closely the estimated force matches the actual applied force. They are presented in Table 4.

(8) $$\mathrm{RMSE}=\sqrt{\frac{1}{N}\sum_{i=1}^{N}{\left(F_{i}-{\hat{F}}_{i}\right)}^{2}}$$

(9) $$R^{2}\left(\%\right)=100\;\left(1-\frac{\sum_{i=1}^{N}{\left(F_{i}-{\hat{F}}_{i}\right)}^{2}}{\sum_{i=1}^{N}{\left(F_{i}-\bar{F}\right)}^{2}}\right)$$

(10) $$\mathrm{Err}\left(\%\right)=\frac{100}{N}\sum_{i=1}^{N}\frac{{\hat{F}}_{i}-F_{i}}{F_{i}}$$

where N is the number of samples, $F_{i}$ the measured load, ${\hat{F}}_{i}$ the load estimated by the model, and $\bar{F}$ the mean value of the measured load.

The same characterization methodology was applied to all sensor nodes of the matrix using an identical experimental protocol. Since the focus of this study is the validation of the sensing principle and characterization procedure, detailed results for the remaining nodes are not included in this paper. Previous investigations conducted on the same sensing technology under dynamic loading conditions with different temporal profiles demonstrated the ability of the inverse viscoelastic model to accurately track force variations over a wide range of operating conditions [48]. However, previous studies were performed on bare sensors and did not consider the integration of the sensing matrix into a textile structure. The present work addresses this limitation by evaluating the model after integration into a wearable glove. The inverse viscoelastic model effectively compensates for the short-term viscoelastic effects associated with the dynamics of the measured motion. In contrast, long-term viscoelastic phenomena require several hours to reach equilibrium, which is several orders of magnitude slower than the time scales involved in paddle gripping tasks. Consequently, long-term effects do not significantly affect force measurements in the considered application.

#### Durability and Recovery Test

**Figure 11:**
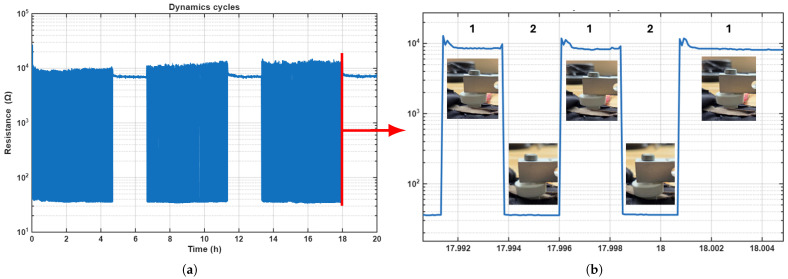
Resistance response of pressure point R7 during the dynamic cycling test: (**a**) complete cycling sequence; (**b**) magnified view of the line highlighted in red in (**a**). State 1 corresponds to a 5 s period without applied pressure (no load), while State 2 corresponds to a 5 s period under applied pressure (loaded).

The results of the durability test are illustrated in Figure 11.

To quantify the influence of cyclic loading, several data values were extracted from each block of 1000 loading cycles. (R_high,init_) denotes the unloaded resistance measured during the first loading cycle, (R_high,mean_) the average unloaded resistance over the 1000 cycles, and (R_high,1000_) the unloaded resistance measured during the 1000th cycle. Similarly, (R_low,mean_) and (R_low,1000_) correspond to the average and final loaded resistances, respectively. Finally, (R_2h,end_) represents the unloaded resistance measured at the end of the 2-h recovery period. The corresponding values are reported in Table 5.

It should be noted that all sensing points of the glove are fabricated from the same piezoresistive material. Consequently, the sensing mechanisms and the associated electromechanical behavior remain similar throughout the sensing array. Although the resistance values may vary between sensing points, the durability and recovery characteristics observed on R7 are representative of the behavior of the other sensing nodes integrated into the glove. Only the low-resistance state was used for force estimation.

### 3.3. Experimentation on an Ergometer

**Figure 12:**
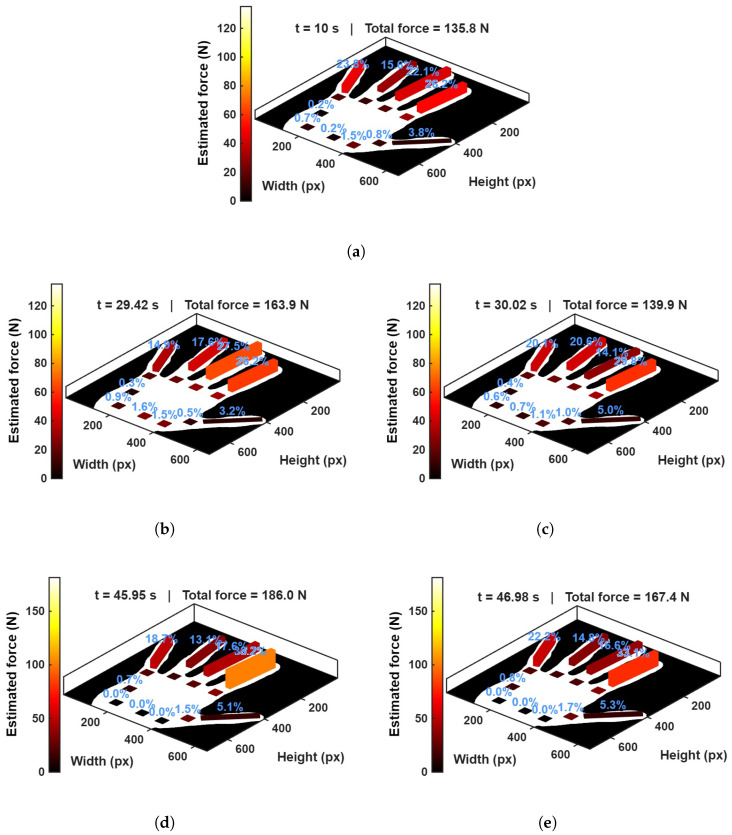
Dynamic estimation of contact forces during the stroke cycle: (**a**) initial position; (**b**) pull phase—moderate cadence; (**c**) push phase—moderate cadence; (**d**) pull phase—fast cadence; (**e**) push phase—fast cadence.

The processing of the experimental results was carried out in Matlab. A 3D mapping of the hand was generated to visualize, as a function of time, the variations of the estimated pressure on each sensor of the matrix during the movements. Each sensor is represented by a vertical bar whose height is proportional to the intensity of the estimated force, expressed as a percentage of the overall calculated force. This overall force is the sum of all sensor values in the matrix. The main results obtained from the dynamic mapping of the experiment are presented in Figure 12. It first illustrates the distribution of pressures when the athlete holds the paddle in a static position before the start of the movement (Figure 12a). This is followed by an initial paddling sequence at a moderate cadence, consisting of a pull phase (Figure 12b), during which the athlete pulls the paddle against the tension applied by the ergometer cords, and a push phase (Figure 12c), during which the athlete accompanies the paddle movement while the left hand pulls on the other side. Finally, these two phases are reproduced at a higher cadence (Figure 12d,e). The maximum values for each phase were plotted in order to highlight the distinct phases observed throughout the motion and to support the proof of concept.

**Figure 13:**
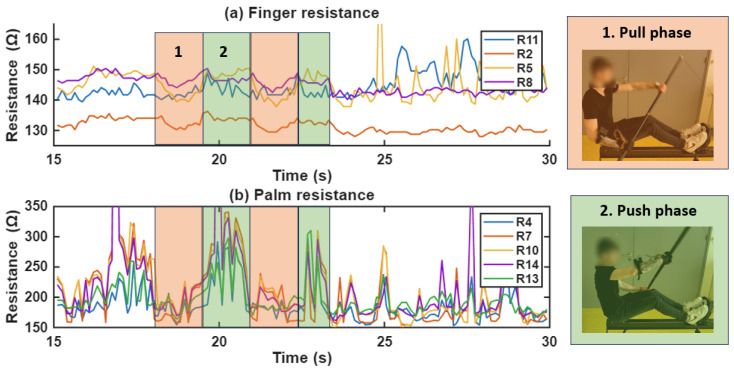
Temporal evolution of representative sensor node resistances during a moderate paddling sequence: (**a**) finger sensor nodes and (**b**) palm sensor nodes. The shaded orange regions correspond to the pull phase (1), while the shaded green regions correspond to the push phase (2).

To complement the pressure maps, the temporal evolution of representative sensor nodes during a moderate paddling cadence is illustrated in Figure 13. The upper graph presents selected nodes located on the fingers, while the lower graph shows representative nodes positioned on the palm. These signals highlight the continuous variation of the sensor responses throughout the paddling cycle and provide the temporal information underlying the pressure distributions presented in Figure 12b,c.

Although these raw signals clearly reflect the dynamic nature of the hand–paddle interaction, their direct interpretation remains challenging because each sensor node exhibits a specific response that depends on its geometry and individual calibration characteristics. This observation highlights the importance of the proposed signal processing and viscoelastic modeling approach, which enables the conversion of resistance measurements into more meaningful load estimates. The lower resistance values observed for the finger sensor nodes compared with those located on the palm are consistent with the higher loads applied to the fingers during paddling, particularly on the index and middle fingers.

## 4. Discussion

Static characterization tests (Figure 9) confirmed good measurement repeatability in high-pressure ranges beyond 10 N, which is consistent with use conditions when gripping the kayak paddle. The low dispersion in these regions (Figure 9a,b) strengthens the reliability of the sensor matrix for dynamic use. This trend was verified at every node of the matrix. The mean relative error obtained from repeated measurements was 3.15 % for the 1 cm^2^ sensors and 1.18 % for the 7 cmv sensors, confirming the good reproducibility of both sensor geometries under static loading conditions. As reported in Table 2, both sensor geometries exhibited a pressure-dependent sensitivity, with higher values obtained in the low-pressure range than in the high-pressure range. The 1 cm^2^ sensors showed greater sensitivity than the 7 cm^2^ sensors, while both configurations exhibited comparable hysteresis levels. This hysteresis does not compromise sensor performance, as it is accounted for and handled by the viscoelastic model used. A decrease in sensitivity was observed as the applied pressure increased, indicating smaller conductance variations for a given pressure increment at higher load levels. This behavior is consistent with the nonlinear response of Velostat and reflects the progressive saturation of the conductive network within the material.

Dynamic characterization (Figure 10) highlighted encouraging performance of the viscoelastic model for estimating load from sensor resistance. The results in Table 4 suggest that the model is suitable for global load estimation, with an RMSE around 2 N and a low mean relative error. However, parameter estimation is required at each node of the matrix. This level of error is not limiting for quantifying large-amplitude phenomena. The dynamic cycling tests presented in Section Durability and Recovery Test demonstrate that the sensor response under load remained highly stable throughout the experiment, with (R_low,mean_) and (R_low,1000_) consistently remaining within a narrow range of approximately 35 Ω to 45 Ω. Although a slight conditioning effect was observed, characterized by a gradual increase in the unloaded resistance during the cycling phase, the resistance returned close to its initial unloaded value after each 2-h recovery period. This behavior indicates that the observed drift is largely reversible and does not originate from permanent degradation of the sensing element. Overall, these results demonstrate that the proposed sensor maintains its functionality and measurement capabilities after several thousand loading cycles under a representative load condition, supporting its suitability for long-term practical use.

The estimated load maps (Figure 12) visualize the spatial and temporal evolution of pressures on the hand over the paddling cycle. During the push and pull phases, there is a clear redistribution of loads across the fingers and the base of the hand. At moderate cadence (Figure 12b,c), loads remain relatively well distributed, with peaks primarily on the fingers, mostly the index and middle fingers, particularly during the pull. At higher cadence (Figure 12d,e), overall load intensity increases, with a more pronounced concentration on the flexor fingers, reflecting greater muscular engagement. Pulling also generates higher loads than pushing, consistent with the paddling mechanics on the ergometer. It should be recalled that the accuracy of the viscoelastic model must be taken into account when interpreting the mapping results.

These results can also be compared with the literature. For example, Hokaria et al. [50] reported a pressure of 250 kPa at the index finger during cylinder-grip experiments. In our high-cadence pull trial, a pressure of 101.5 kPa was recorded, which is of the same order of magnitude and supports the consistency of our findings with prior reports. Moreover, Bonito et al. [25] reported force peaks averaging up to 240 N at each hand in elite kayakers. Our measurements indicate total loads reaching 186 N during the most intense phases, which is consistent with laboratory conditions, even though the cadence here (up to 48 strokes/min) remains lower than peak race cadences. It should also be noted that the participant involved in this study was not a high-level athlete. In the study by Niu et al. [51], competitive female kayakers reached a mean right-hand grip force of 100.55 N during right-side strokes.

It should be noted that this experiment was primarily intended to validate the operation of the instrumented glove. The values obtained open promising avenues for assessing technique and conducting biomechanical analysis in a controlled environment.

## 5. Conclusions

The study enabled the development and experimental validation of an instrumented glove for elite kayaking, integrating a matrix of piezoresistive sensors coupled with a viscoelastic model to estimate the forces applied by the hand to the paddle throughout the movement. The results demonstrate the model’s accuracy, as well as good consistency of the dynamic maps with the different movement phases observed in ergometer tests and in the literature. Although this study is preliminary and based on a limited number of trials, it establishes a proof of concept. An energy assessment of the system, which is not detailed here, showed an autonomy of 26 h, which is more than sufficient for a training session with high-level athletes.

Although Velostat has been widely adopted in the literature for pressure-sensing applications, reported models, when provided, are most often restricted to static load or force estimation. In this work, we go beyond this perspective by leveraging a dynamic model that has already been validated in the literature [48], and we observe behavior that is consistent with previously published results. This validated framework then supports the definition and demonstration of new application scenarios, notably through the integration of stitched Velostat into textile-based structures, as presented in this paper.

There are many avenues for future work. On the hardware side, it would be valuable to ensure that the glove is fully waterproof for on-water testing and to characterize sensor stability under real humidity and temperature conditions. This includes characterizing not only the sensor itself but also the rubber-based waterproofing material used in wetsuits, as its viscoelastic behavior may influence the overall performance and reliability of the system. Developing a glove for the other hand would enable bilateral analysis of the stroke. For data transmission, a long-range communication system would allow coaches to perform real-time monitoring. Investigating the long-term mechanical behavior of the sensing material, including potential creep, stress relaxation, and fatigue effects, would be valuable to assess whether these phenomena could influence sensor performance and the reliability of the measured data over extended periods of use. Finally, combining these force data with variables such as stroke cadence or boat speed would offer new indicators of performance and individual technique.

This work has cross-cutting value for stakeholders across sports. Athletes would benefit from objective feedback on their technique, coaches would benefit from a tool to support technical monitoring, researchers would benefit from a reusable platform for movement analysis or load assessment, and the medical sector would benefit from a device potentially transferable to functional rehabilitation. Access to these measurements would also make it possible to identify movement asymmetries that may affect other parameters, such as stroke rate. In addition, quantifying the effort and combining it with other biomechanical and physiological indicators would enable a more precise assessment of its contribution to boat speed. At present, boat speed is typically measured independently of these parameters; estimating speed as a function of multiple metrics would provide clearer guidance for training, improve athlete monitoring, and enhance performance. Future work could combine the pressure measurements provided by the glove with other biomechanical and physiological indicators to enable a more comprehensive assessment of athlete performance. The system, therefore, paves the way for a compact, customizable tool.

## Figures and Tables

**Table 1:** Commercial instrumented glove systems available on the market.

Systems	Applications	Transmission	Sampling Rate	Sensing Elements	Sensor Thickness (mm)	Pressure Range
Grip™ [28]	Pressure mapping	USB/Wi-Fi	750/200 Hz	349	0.15	0–345 kPa
Data Glove Ultra [29]	Finger flexion	USB/Bluetooth	2.4 GHz	5–14	/	/
Tactile Glove [30]	Pressure mapping	Bluetooth (BLE)	25–40 Hz	65	2.6	80 psi
Carbonhand [31]	Finger pressure	Bluetooth	2.5 GHz	6	/	20 N
Tactilus [32]	Finger pressure	Bluetooth	1 Hz	4	5	0–100 psi

**Table 2:** Static characterization of the two sensor sections.

Surface (cm^2^)	*S*_*low*_ (μS/kPa)	*S*_*high*_ (μS/kPa)	Hysteresis_*max*_ (%)	Hysteresis_*mean*_ (%)
1	1.20	0.26	24.97	9.83
7	0.26	0.05	21.80	9.15

**Table 3:** Optimized coefficients of the inverse viscoelastic model.

Parameter	1 cm^2^ Sensor	7 cm^2^ Sensor
$E_{0a}$ (Pa/N)	$1.38\times 10^{5}$	$1.06\times 10^{5}$
$E_{0b}$ (Pa)	$2.79\times 10^{6}$	$3.12\times 10^{6}$
$E_{1a}$ (Pa/N)	$1.00\times 10^{4}$	$3.00\times 10^{3}$
$E_{1b}$ (Pa)	$7.17\times 10^{6}$	$7.39\times 10^{6}$
$\mu_{1a}$ (Pa.s/N)	$3.06\times 10^{4}$	$5.55\times 10^{4}$
$\mu_{1b}$ (Pa.s)	$1.41\times 10^{6}$	$6.90\times 10^{5}$

**Table 4:** Viscoelastic model performance for both sensor sections.

Surface (cm^2^)	R^2^ (%)	RMSE (N)	Mean Relative Error (%)
1	69	2.12	2.72
7	91	2.27	2.44

**Table 5:** Durability and recovery performance of sensing point R7 during cyclic loading tests.

Cycle	$R_{\mathrm{high},\mathrm{init}}$ (Ω)	$R_{\mathrm{high},\mathrm{mean}}$ (Ω)	$R_{\mathrm{high},1000}$ (Ω)	$R_{\mathrm{low},\mathrm{init}}$ (Ω)	$R_{\mathrm{low},1000}$ (Ω)	$R_{2h,\mathrm{end}}$ (Ω)
1	7016.3	7703.3	8343.5	44.2	35.6	6884.3
2	6930.2	8090.7	8480.0	37.7	36.6	7036.9
3	6946.4	8448.0	8813.3	39.3	35.9	7192.4

## Data Availability

The data presented in this study are available on request from the corresponding author.

## Abbreviations

The following abbreviations are used in this manuscript:

BLE	Bluetooth Low Energy
RMSE	Root Mean Square Error
FSR	Force-Sensing Resistor
EMG	Electromyography
PVDF	PolyVinyliDene Fluoride
IDC	Insulation Displacement Connector
PLA	Polylactic Acid
FFCK	French Canoe Kayak Federation
